# Is cystatin C reliable in the anesthetized pig? An experimental study with special reference to septic shock

**DOI:** 10.1186/cc10962

**Published:** 2012-03-20

**Authors:** M Eriksson, E Söderberg, M Lipcsey, J Sjölin, M Castegren, M Sjöquist, A Larsson

**Affiliations:** 1 Surgical Sciences, Anesthesia & Intensive Care, Uppsala, Sweden; 2 Medical Sciences, Infectious Disease, Uppsala, Sweden; 3 Centre for Clinical Research, Eskilstuna, Sweden; 4 SLU, Uppsala, Sweden; 5 Medical Sciences, Clinical Chemistry, Uppsala, Sweden

## Introduction

Our aim was to investigate renal function during 24 hours of endotoxemic shock with special focus on the reliability of analysis options in kidney damage.

## Methods

Twenty anesthetized pigs received randomly a continuous 24-hour endotoxin infusion at 0.063 μg/kg/hour (*n *= 8) or 0.25 μg/kg/hour (*n *= 9) or NaCl (controls *n *= 3). Boluses (10 ml/kg) of succinylated gelatin were given when the arterial blood pressure was 50 mmHg or below. Samples for analysis of cystatin C as well as clearances of inulin, PAH and creatinine were noted and urine was collected.

## Results

Cystatin C was, already at baseline, not normally distributed. This was in contrast to the other renal variables. Five pigs had baseline values of cystatin C in plasma >0.6 mg/l (one control; four endotoxemic pigs), whereas 15 pigs had plasma levels < 0.3. When individual values were noted over time, it became obvious that, with the exception of the four endotoxemic animals, which shifted considerably over time, there appears to be two subgroups of pigs regarding their cystatin C values. There were only minor differences in cystatin C over time for each individual pig compared with the baseline value, except for the four pigs that shifted considerably over time. There were no major differences in urinary output between untreated controls and any of the two endotoxemic groups of pigs during the 24-hour experimental period. There was no obvious relation between the administration of bolus doses of gelatin and subsequent diuretic response. Cystatin C did not correlate to creatinine clearance (*r*^2 ^= 0.06), PAH clearance (*r*^2 ^= 0.05), inulin×urine (*r*^2 ^= 0.04) or diuresis (*r*^2 ^= 0.004). No similar subgroupings were noted for any of the other renal variables, although it should be noted that correlations between all variables were weak.

## Conclusion

In this experiment, we noted that there appears to be two populations of pigs regarding their cystatin C values. This result is in contrast to a previous study from our group [1]. Our findings may be explained by the alterations that occur in renal vascular resistance [2], although these findings may also indicate a genetic variation influencing either the levels of cystatin C or the antigen determinants of cystatin C. Until our data have been confirmed or disproved, we strongly suggest that porcine Cystatin C values should be interpreted with great care as a marker for glomerular filtration rate in pigs.
